# Application of Cu/Mg/Al-chitosan-O_3_ system for landfill leachate treatment: Experimental and economic evaluation data

**DOI:** 10.1016/j.dib.2017.07.063

**Published:** 2017-07-27

**Authors:** Dariush Ranjbar Vakilabadi, Bahman Ramavandi, Amir Hessam Hassani, Ghasemali Omrani

**Affiliations:** a Department of Environmental Science, Faculty of Environment and Energy, Science and Research Branch, Islamic Azad University, Tehran, Iran; b Environmental Health Engineering Department, Faculty of Health, Bushehr University of Medical Sciences, Bushehr, Iran; c Department of Environmental Engineering, Faculty of Environment and Energy, Science and Research Branch, Islamic Azad University, Tehran, Iran

**Keywords:** Landfill leachate treatment, Cu/Mg/Al-chitosan, Chemical oxygen demand, Colour, Economic evaluation

## Abstract

Landfill leachate contains heavy organic pollutants, which pollute ground and surface waters. This dataset applied a newly-introduced catalyst, Cu/Mg/Al-chitosan, for a landfill leachate treatment during a catalytic oxidation. The data of chemical oxygen demand (COD) and colour removal from the leachate was reported as a function of reaction time (20–460 min). Economic evaluation data of the Cu/Mg/Al-chitosan-O_3_ system showed that the current cost of the system for treating each m^3^ leachate is US$ 18 and for catalyst synthesis is US$ 54.5. Data could be useful from environmental and economic perspectives to those concerned about landfill leachate threats.

## Specifications Table

**Table:** 

Subject area	*Chemical engineering*
More specific subject area	*Environmental engineering; Wastewater treatment*
Type of data	*Figure and table*
How data was acquired	*The landfill leachate pH was determined using a Jenway 3505 pH meter. The COD analysis was conducted using the potassium dichromate oxidation method.*
Data format	*Analysed*
Experimental factors	– *Cu/Mg/Al-chitosan particles were provided by the precipitation method.* – *Landfill leachate sample was treated in a given reaction time as a function of COD and colour.* – *The kinetic of the leachate treatment was determined.* – *The economic evaluation for Cu/Mg/Al-chitosan-O*_*3*_ *was presented.*
Experimental features	*Landfill leachate treatment by Cu/Mg/Al-chitosan-O*_*3*_*system*
Data source location	*Bushehr University of Medical Sciences, Bushehr, Iran, GPS: 28.9667°N, 50.8333°E*
Data accessibility	*Data presented with article*

## Value of the data

• A new catalytic process for landfill leachate treatment was introduced to the scientific community.
• From our data, it could be implied that the COD was decreased to discharge allowable limit to wastewater collection system, compared to biological leachate treatment systems.
• Data shows that the Cu/Mg/Al-chitosan-O_3_ system is an economic process for landfill leachate treatment.
• Many organizations like waste management organizations, wastewater treatment plants, water resources management, NGOs, etc., which are concerned about the hazards from landfill leachate, can use these data.

## 1 Data

**Fig. 1:**
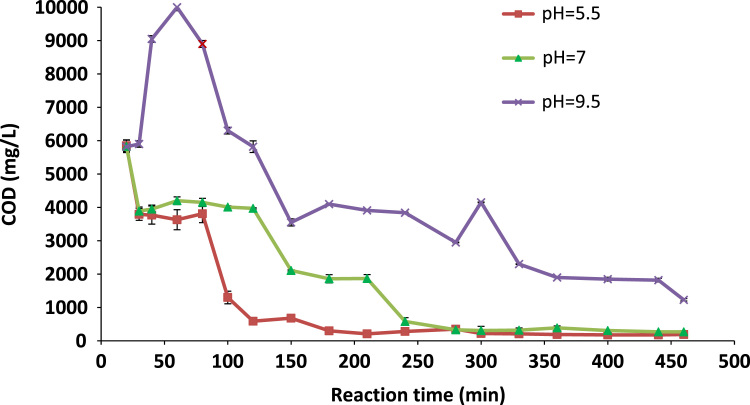
Final COD of the treated leachate at different pH.

**Fig. 2:**
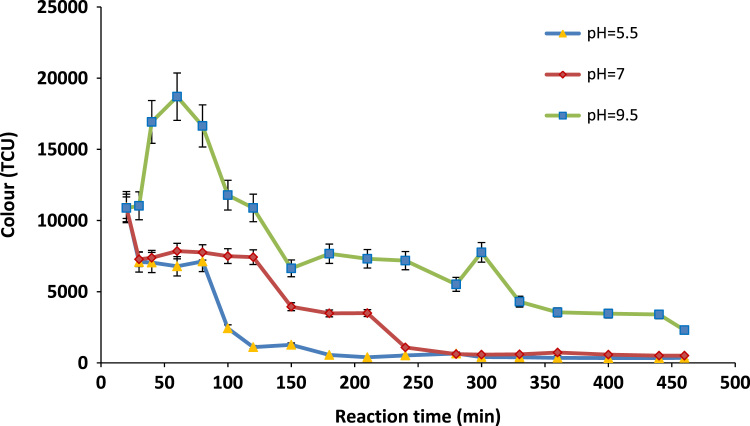
Final color of the treated leachate at different pH.

**Fig. 3:**
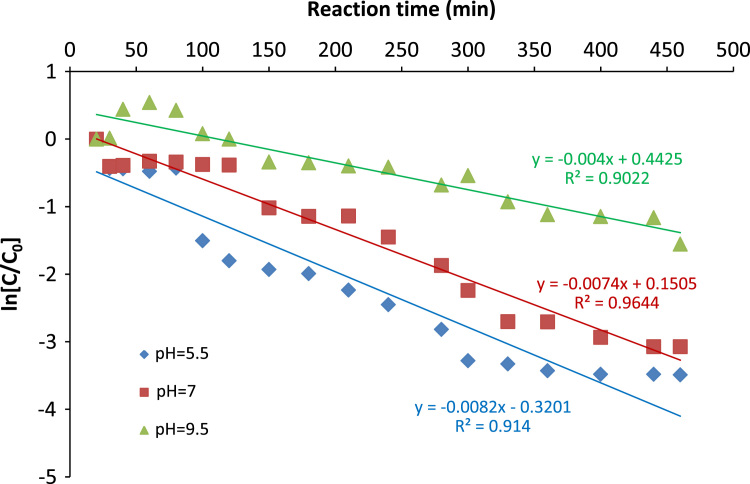
Pseudo first order kinetic plot for the COD degradation in batch experiments.

Table 1 shows the characteristics of the raw leachates. Fig. 1, Fig. 2 depict the COD and colour removal at different leachate pHs respectively as a function of time. Fig. 3 shows the pseudo first-order kinetic plot for the leachate COD removal by the Cu/Mg/Al-chitosan-O_3_ system. Results for the economic evaluation of leachate treatment by Cu/Mg/Al-chitosan-O_3_ system are presented in Table 2.

**Table 1:** Raw landfill leachates characteristics.

Property	Value±SD		
	Sample 1	Sample 2	Sample 3
pH	9.5±0.3	7±0.3	5.5±0.2
Colour	Blackish brown	Black	Brown blackish
COD (mg/L)	40,700±44	25,009±39	1938±73
TOC (mg/L)	34,040±46	15,500±69	16,700±86
BOD_5_ (mg/L)	2100±36	1800±41	1830±67
BOD_5_/COD ratio	0.052	0.072	0.11
Alkalinity (mg/L CaCO_3_)	10,078±26	8955±34	6790±23

**Table 2:** Assumptions and results for the economic evaluation.

Case	Assumptions (value and unit)	Cu/Mg/Al-chitosan production cost (US$/kg)	Leachate treatment by Cu/Mg/Al-chitosan-O_3_ (US$/m^3^)
Electricity cost (industrial)	121.46 US$/MWh	5	2
Heat cost	36.1 US$/MWh	0.5	–
HCl price	660 US$/ton	5	–
NaOH price	330 US$/ton	4	–
Mg(NO_3_)_2_ price	250 US$/ton	6	–
Al(NO_3_)_3_ price	5000 US$/ton	14	–
Cu(NO_3_)_2_ price	250 US$/ton	5	–
KI price	220 US$/ton	–	6
Chitosan price	15,000 US$/ton	15	–
Ozone generator price	300 US$	–	10
General cost (US$)		54.5	18

The data of Refs. [1], [2] were used in this table.

## 2 Experimental design, materials and methods

### 2.1 Landfill leachate sampling

Leachate samples of a municipal solid waste landfill were collected with 5 l-polyethylene bottles from Kahrizak, Tehran, Iran during the summer of 2015 and transferred in cooler boxes at a temperature below 5 °C to the laboratory within 2 h. The landfill leachates were sampled from different leachate ponds with different pHs (9.5, 7, and 5.5). All samples were taken from the surface of leachates. Leachate samples were stored in a refrigerator at 4 °C before tests. The physicochemical characteristics of the raw leachates are listed in Table 1.

### 2.2 Catalyst preparation

The catalyst in this dataset was Cu/Mg/Al-chitosan (CMA-chitosan), which was prepared by the precipitation method. Details about the preparation of the catalyst have been reported elsewhere [2], [3].

### 2.3 Test procedure

A reactor with working volume of 300 mL, equipped with an ozone generator (Model 3S-A3, Tonglin Technology, Beijing), a sintered diffuser to evenly distribute the ozone stream to the landfill leachate, an air pump, KI solution as ozone off-gas destructor, valves, and tubing. The O_3_-generator capacity was 5 g/h. The O_3_ flow rate was set to 3.5 mg/min throughout the experiments. About 200 mL of landfill leachate with original COD and pH value (see Fig. 1, Fig. 2) was poured in the reactor and the final COD was analysed at reaction time of 20–460 min. All tests were done with three repetitions under room temperature (24±1 °C) and atmospheric condition and average values beside standard deviation (SD) were reported herein.

### 2.4 Measurements

The landfill leachate pH was determined on site at the time of sampling, using a digital pH meter. The COD measurements were done using the potassium dichromate oxidation method [4]. Leachates BOD_5_ was determined in accordance with the 5210-D test using manometric respirometry (OxiTop) [4]. A total organic carbon (TOC) analyser (Shimadzu Co.) was applied to determine TOC content of leachates. Other characteristics of the leachates were recorded in accordance with the standard methods for the examination of water and wastewater [4].
