# Supplementary material for: Application of Cu/Mg/Al-chitosan-O3 system for landfill leachate treatment: Experimental and economic evaluation data
Source: Data Brief. 2017 Jul 27;14:192–6. doi: 10.1016/j.dib.2017.07.063 (PMC5537446; doi:10.1016/j.dib.2017.07.063)
Supplement: Supplementary file 1 — Supplementary material [file mmc1.docx]

**Conflict of interest**

The authors declare no conflict of interest associated with this data article.
